# microRNA network analysis identifies miR-29 cluster as key regulator of LAMA2 in ependymoma

**DOI:** 10.1186/s40478-015-0206-2

**Published:** 2015-05-10

**Authors:** Anbarasu Lourdusamy, Ruman Rahman, Stuart Smith, Richard Grundy

**Affiliations:** Children’s Brain Tumour Research Centre, School of Medicine, Queen’s Medical Centre, University of Nottingham, Nottingham, NG7 2UH UK

**Keywords:** microRNA, Ependymoma, Regulatory network, Microarray

Ependymomas (EPN) are enigmatic tumors which continue to present significant management challenges to clinicians as evidenced by the failure to cure up to 40% of cases [1]. Recent genomic and epigenomic studies have identified alterations in DNA copy number, gene expression [2,3], and methylation [4] and showed that EPN is a heterogeneous disease and consists of distinct molecular subtypes. However, the involvement of the microRNAs (miRNA) and their influence over mRNA translation into proteins, in EPN and their contribution to the complexity of the disease are still poorly understood.

To identify miRNA - mRNA regulatory network, we systematically evaluated miRNA - mRNA associations using expression profiles of tumors from 64 EPN patients (mean age of 13.3 years, Additional file 1: Table S1 for more details). For each miRNA-mRNA pair, we measured the association between miRNA and mRNA using a Spearman rank correlation and filtered with sequence-based predicted miRNA-target interactions of miRanda and TargetScan databases Additional File 3: Materials and methods [5,6]. We selected miRNA-mRNA pairs with strong negative correlation (FDR < 0.005, 78,934 pairs) and evidence for target interaction as predicted by miRanda (score < −0.5), TargetScan (context score < −0.2) and evolutionary conservation (miRanda conservation score > 0.5). We used these thresholds to obtain a high-confidence list of candidate miRNA-target interactions. The combination of the correlation and target prediction filters yielded 390 miRNA-mRNA pairs, significantly more than was expected by chance (*P* = 5.95 × 10^−08^, two-tailed binomial test, *x* = 390, *n* = 78,934, *p* = 3.71 × 10^−03^ = 28,309 predicted targets/7,623,897 total pairs). These 390 putative target interactions from EPN consisted of 107 evolutionarily conserved miRNAs and 305 target mRNAs (Additional file 1: Table S2). Remarkably, these miRNAs are significantly enriched for oncomiRs/tumor suppressor miRNAs (2.01 fold enrichment; P = 9.41 × 10^−03^, Fisher’s exact test. Additional file 2: Figure S1). They included let-7c, miR-125b, miR-29a/b, miR-15 family – miR-15a, miR-16, and miR-196 (tumor suppressors miRNA), miR-18a/b, miR-19a/b, and miR-17 family – miR-106a/b, miR-17, miR-20a/b, and miR-93 (onco miRNA). Growing evidences suggest that miR-17 miRNAs are involved in cell proliferation, development, and stem cell differentiation [7]. In addition, members of miR-17 directly target *TGFBR2*, attenuate *TGF-β* signaling that regulates multitude of cellular processes, and is particularly relevant not only during development, but also in cancer initiation and progression [7,8]. Targets genes regulated by miRNAs are enriched for biological processes or pathways such as multicellular organismal development (GO:0007275; FDR = 3.31 × 10^−04^), neuron differentiation (GO:0030182; FDR = 3.85 × 10^−04^), regulation of cell adhesion (GO:0030155; FDR = 3.75 × 10^−03^), MAPK signalling pathways (hsa04010; FDR = 2.67 × 10^−03^), pathways in cancer (hsa05200; FDR = 3.60 × 10^−03^), extracellular matrix (ECM) receptor interaction (hsa04512; FDR = 1.22 × 10^−02^), and Integrin, EGF receptor, Wnt, and Neurotrophin signaling pathways (Additional file 1: Table S3 ). Enrichment of known cancer-related processes and pathways among miRNAs and target genes suggest that inferred miRNA-target relationships have functional roles in EPN.

The inferred putative target interactions from EPN formed a network with 11 highly interconnected subnetworks (Additional file 2: Figure S2). At least 50 of the 390 EPN target interactions (comprising 25 miRNAs and 35 mRNAs) have strong evidence of recurrence in multiple cancer types (recurrence score < −3.0 and FDR < 0.05) on the basis of The Cancer Genome Atlas (TCGA) miRNA-target interactions analysis across 10 cancer types (Figure 1, Additional file 1: Table S2) [9]. EPN interactions with high recurrence score include pairs such as miR-18a:*CREBL2* (REC score = −10.56; FDR = 1.81 × 10^−07^), miR-106b:*TGFBR2* (REC = −9.07; FDR = 2.52 × 10^−06^), and the interactions between the miR-29 family and *NREP* (REC = −19.74; FDR = 7.05 × 10^−15^), *UBTD2* (REC = −9.97; FDR = 4.99 × 10^−07^), and *MEX3B* (REC = −5.86; FDR = 6.64 × 10^−04^) that physically interacts with the *AGO1* gene, encoding a member of the Argonaute family of proteins which play a role in RNA interference by miRNAs via the RISC complex. The network also showed several novel EPN-specific interactions with genes that are involved in axon guidance (miR-29a:*LAMA2,* miR-29c:*LAMA2),* histone H3-K27 methylation (miR-106B:*EZH1*), chromatin modification (miR-29c:*JARID2*), and methylation-dependent chromatin silencing (miR-29a:*DNMT3B*).

**Figure 1:**
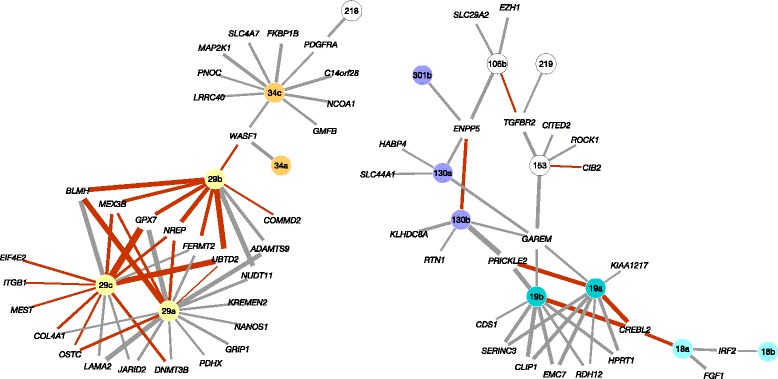
Ependymoma network of miRNA-mRNA target interactions. Two highly connected sub-networks from the inferred network of ependymoma comprising 390 putative target interactions between 107 miRNAs and 305 target mRNAs. Edge width represents strength of Spearman rank correlation for a given miRNA-mRNA pair, red edge colour represents evidence of recurrence in multiple cancer types, and miRNAs from the same family are color coded while a single miRNA is shown in white.

**Figure 2:**
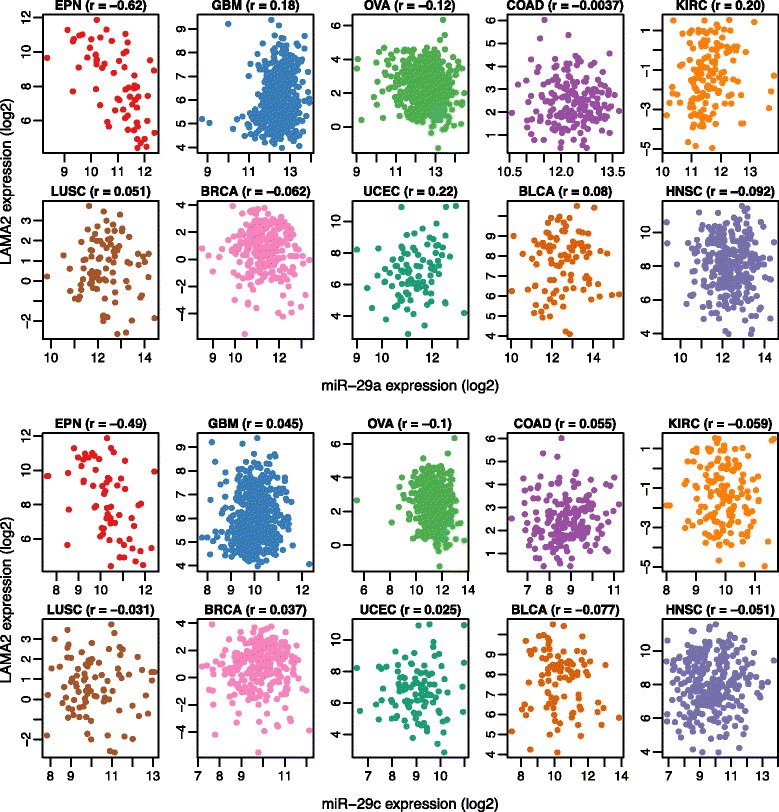
miR-29 family regulation of posterior fossa subgroup marker *LAMA2*. The relationship between *LAMA2* mRNA expression and miR-29a (left) and miR-29c (right) expression in tumor samples from ten different cancer types using Spearman rank correlation, showing strong association only in EPN. EPN: Ependymoma; GBM: Glioblastoma multiforme; OVA: Ovarian serous cystadenocarcinoma; COAD: Colon and rectal adenocarcinoma; KIRC: Kidney renal clear cell carcinoma; LUSC: Lung squamous cell carcinoma; BRCA: Breast invasive carcinoma; UCEC: Uterine Corpus Endometrioid Carcinoma; BLCA: Bladder Urothelial Carcinoma; HNSC: Head and Neck squamous cell carcinoma.

Next, we asked whether the network represented by these putative target interactions captures the salient transcriptomic features of EPN subgroups, particularly from posterior fossa (PF). In line with earlier studies, a consensus clustering analysis of mRNA expression data among 64 EPN cases led to the identification of three transcriptional subtypes: largely supratentorial (ST, 37.5%), PF with spinal (SP) and ST (PF + SP + ST, 29.7%), and largely PF (PF, 32.8%) (Additional file 2: Figure S3). The group largely associated with PF (named as PFA) comprised predominantly of children with mean age of 3.5 years (range from 0.4 – 9.2 years), whereas the PF clustered with SP and ST (named as PFB) consisted of adults with a mean age of 24.5 years (range from 8 – 45 years) (Additional file 2: Figure S4). The differential expression analysis between the two PF subgroups detected a total of 46 mRNA targets as significant (FDR < 0.05), and 27 of these were also differentially expressed in an independent dataset (P = 6.20 × 10^−05^, Fisher’s exact test) of 37 PF EPN from the previously published study (Additional file 1: Table S4). Remarkably, the direction of expression differences between two PF subgroups was same in both cohorts for all of the 27 overlapping differentially expressed genes (Additional file 2: Figure S4). Fifteen overlapping mRNA target genes showed high levels of expression in the PFA subgroup, of which four (*ACSL6*, *ATP1B2*, *CAMK2B*, *and S1PR1*) were regulated by miR-24, four (*ADAMTS9*, *BLMH*, *LAMA2*, and *NUDT11*) were regulated by the miR-29 family, three (*MMD*, *PKIA*, and *STK39*) were regulated by miR-27b, and *PDGFRA* was regulated by miR-34c and miR-218. Interestingly, *LAMA2* has previously been identified as a candidate maker gene for PFA subgroup and associated with poor prognosis [3,10,11]. In addition to a strong miR29a:*LAMA2* association (*r* = −0.62; FDR = 1.18 × 10^−05^), we also observed an inverse correlation between *LAMA2* and miR29c (*r* = −0.49; FDR = 1.61 × 10^−03^) in EPN, but not in other cancer types (Figure 2). The two miR-29 family miRNAs are encoded at two different genomic loci, yet they showed anti-correlation with *LAMA2* in EPN, suggesting EPN-specific strong co-regulation of the miR-29 loci. Taken together, these results suggest that downregulation of miR-29 family expression is a potent mechanism by which *LAMA2* expression is altered in PF ependymoma.

In summary, we identified miR-29a/c as novel regulators of *LAMA2* in ependymoma based on miRNA-mRNA covariation and sequence-based target predictions. The decreased expression of miR-29a/c and elevated *LAMA2* expression are therefore defining features of PF ependymoma post-transcriptional regulation, indicating a key mechanism for molecular pathogenesis. Apart from changes in miRNA expression, other mechanisms (genetic or epigenetic) can contribute to *LAMA2* expression in PF ependymoma, which now mandates further investigation.

## Additional files

Additional file 1: Table S1. Characteristics of ependymoma patients used in the current study. **Table S2.** Putative miRNA – target interactions from Ependymoma expression data. **Table S3.** Enrichment of biological processes and pathways. **Table S4**. Differential expression of miRNA target genes in PF subgroups. Additional file 2: Figure S1. miRNAs in the miRNA-target network are enriched for oncomir and tumor suppressor miRNAs. **Figure S2.** Inferred miRNA-target network of 390 putative target interactions is clustered into 11 highly interconnected sub networks. **Figure S3.** Performance of consensus clustering showing optimum partitioning of three ependymoma (EPN) subtypes. **Figure S4.** Characteristics of two PF subgroups (difference in Age, left and consistent difference in gene expression). Additional file 3:
**Materials and methods.**

## Abbreviations

EPN: Ependymoma
miRNA: micro RNA
ECM: Extra cellular matrix
TCGA: The Cancer Genome Atlas
PF: Posterior fossa
ST: Supratentorial
SP: Spinal
PFA: Posterior fossa subgroup A
PFB: Posterior fossa subgroup B
